# Supplementary figures and images for: Dopamine neuron morphology and output are differentially controlled by mTORC1 and mTORC2
Source: eLife. 2022 Jul 26;11:e75398. doi: 10.7554/eLife.75398 (PMC9328766; doi:10.7554/eLife.75398)

DA-Raptor KO  
Western blots  
Gel 1

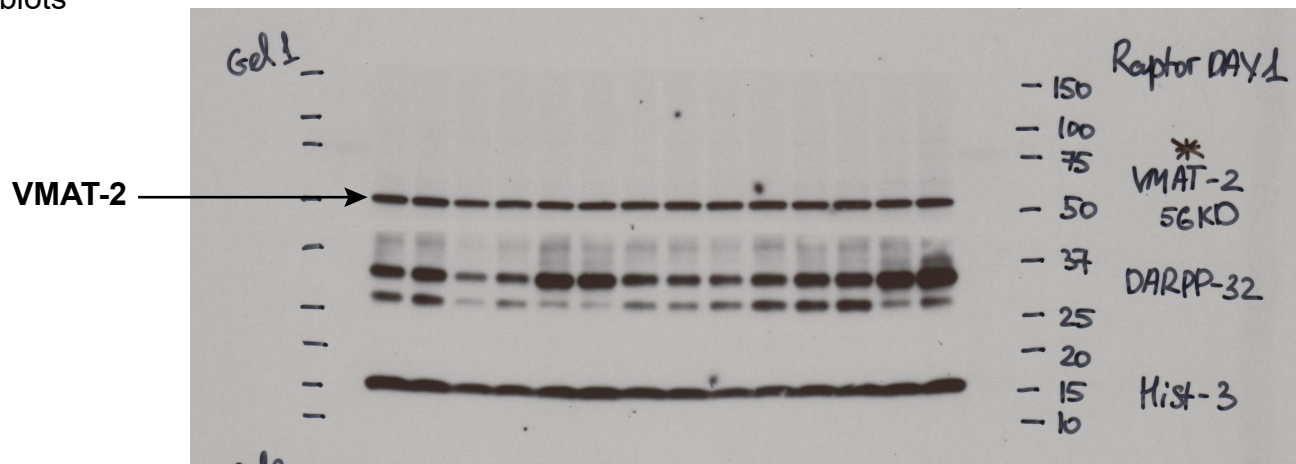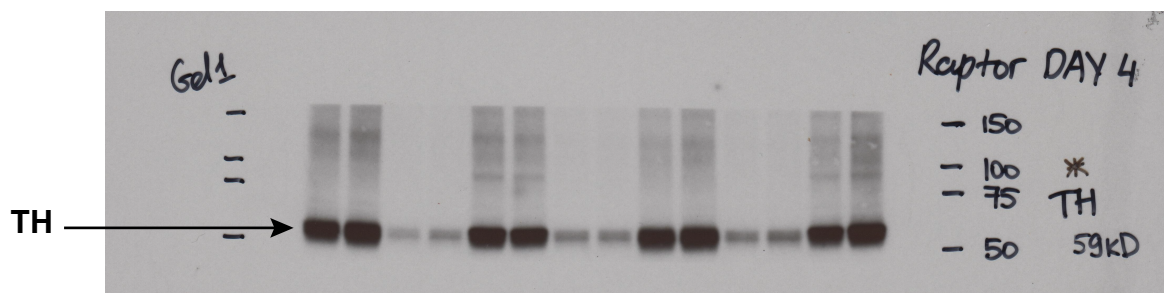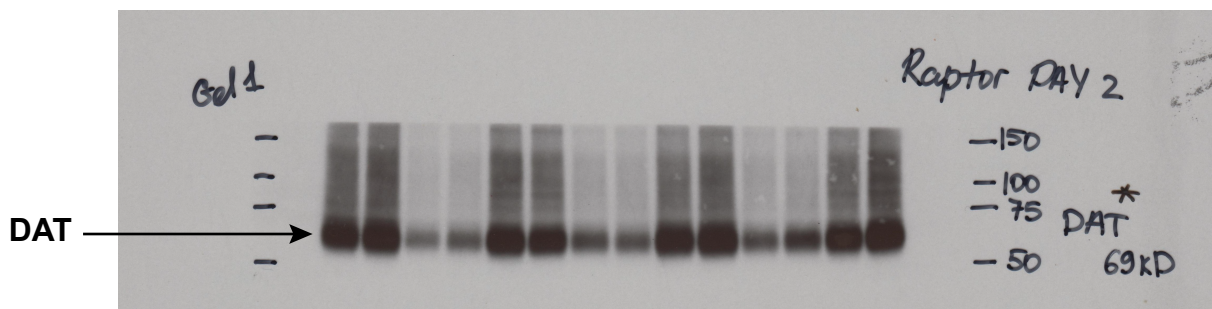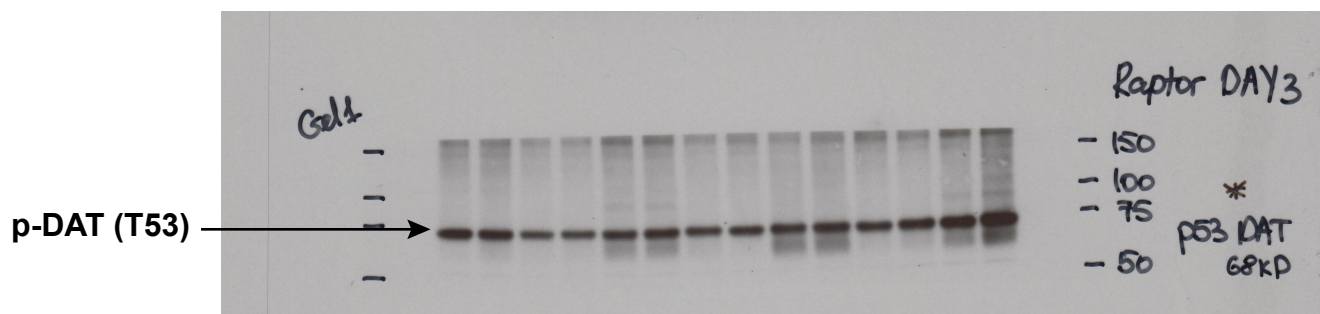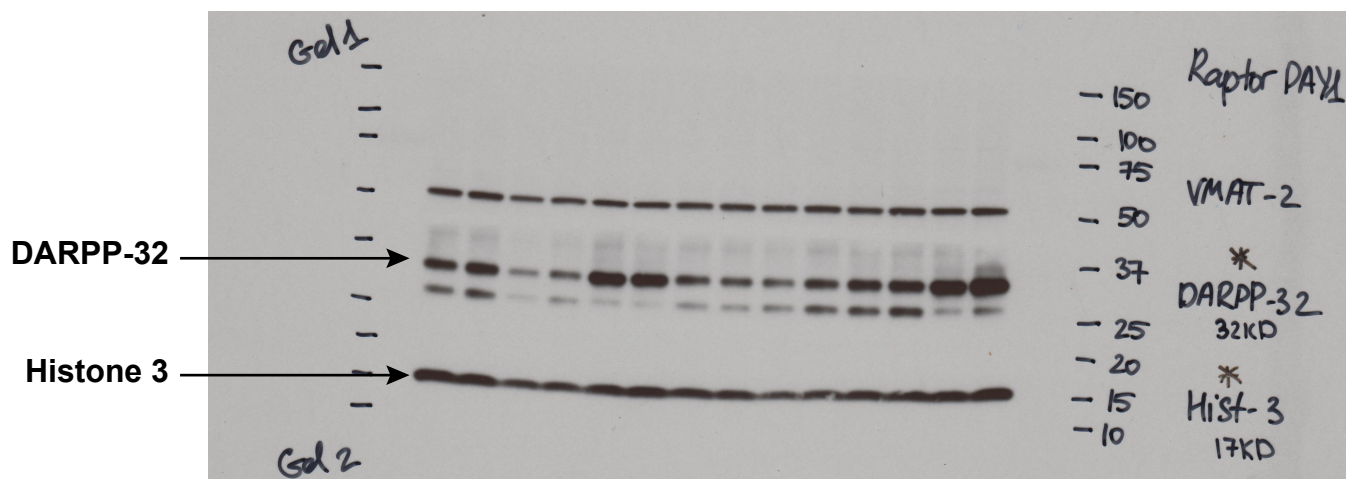

Supplement: Figure 8—source data 1. — The scans used for quantification for each antibody are indicated by the arrows. Films containing the first batch of samples (Gel 1) are shown. [file elife-75398-fig8-data1.pdf]

DA-Raptor KO  
Western blots  
Gel 2

VMAT-2

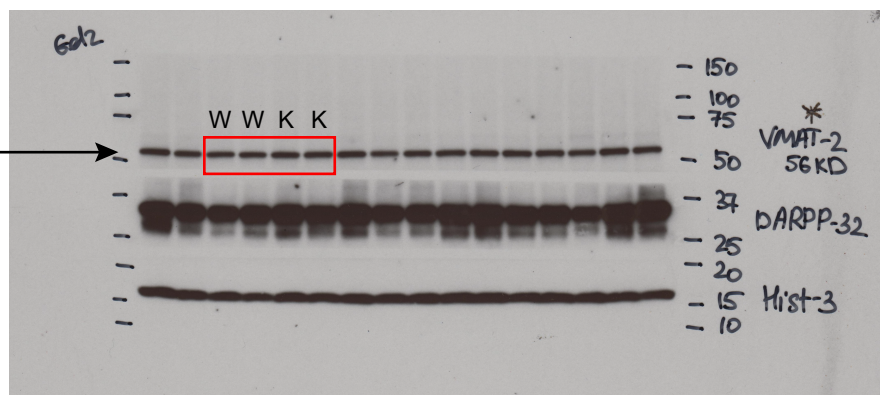

TH

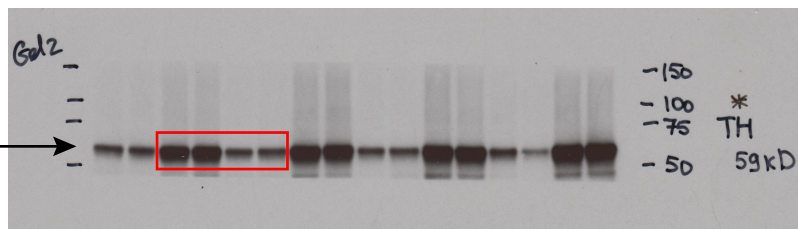

DAT

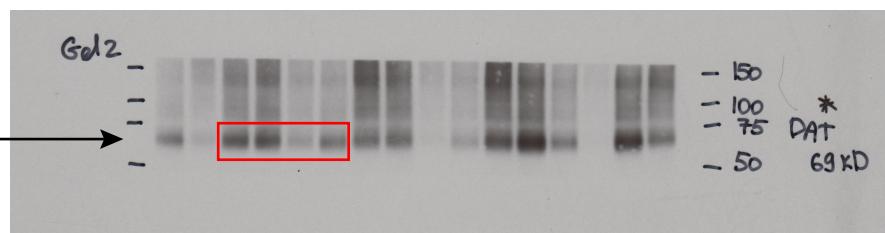

p-DAT (T53)

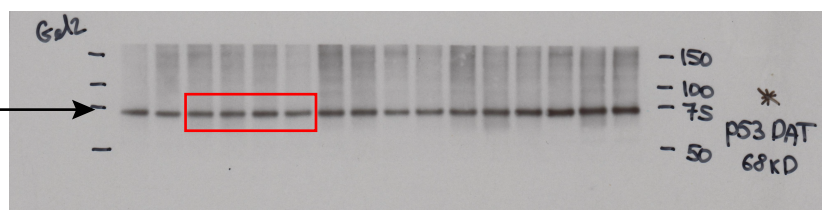

DARPP-32

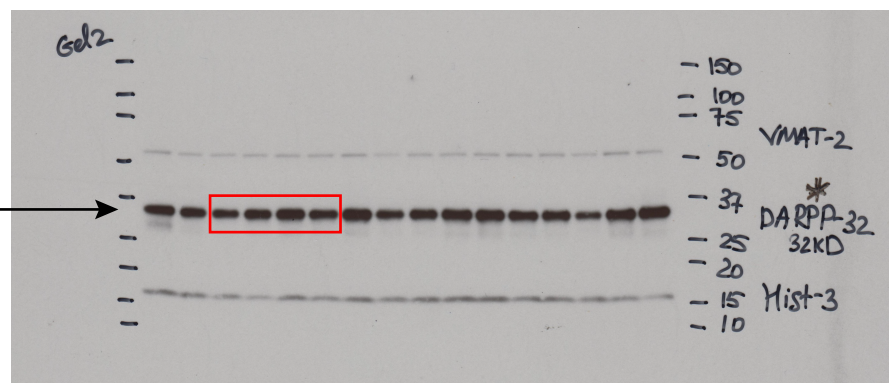

Histone 3

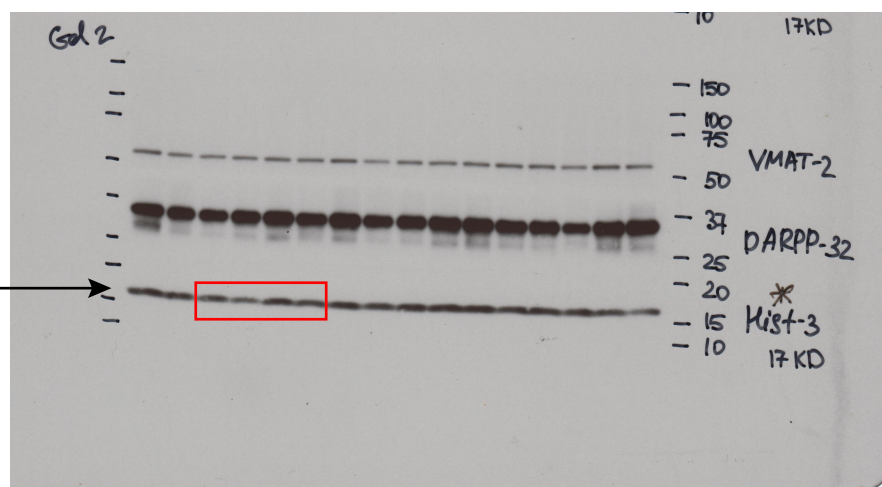

Supplement: Figure 8—source data 2. — The scans used for quantification for each antibody are indicated by the arrows. Films containing the second batch of samples (Gel 2) are shown. Shown in red are the cropped regions presented in Figure 8a. W=wild type, K=knock out. [file elife-75398-fig8-data2.pdf]

DA-Rictor KO  
Western blots  
Gel 1

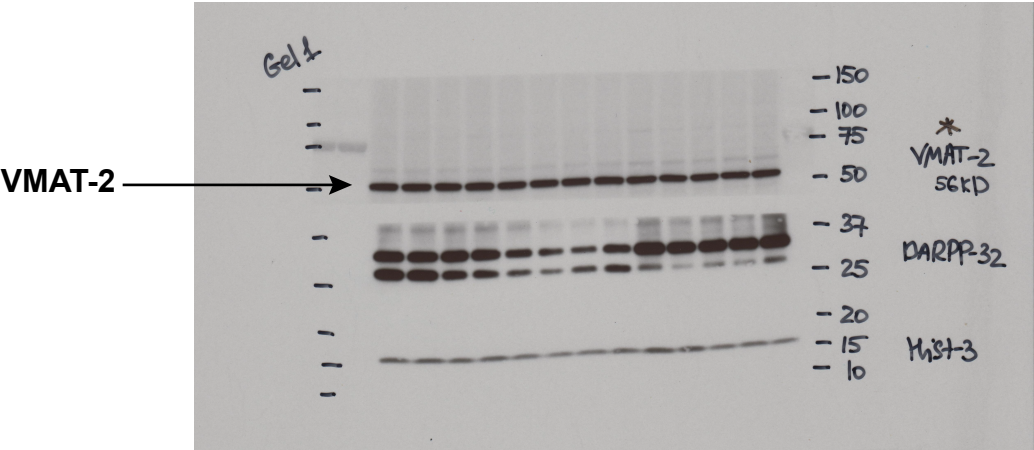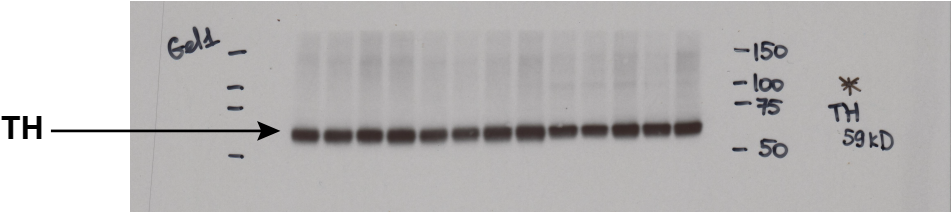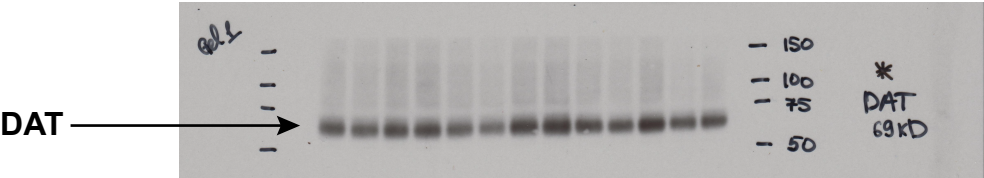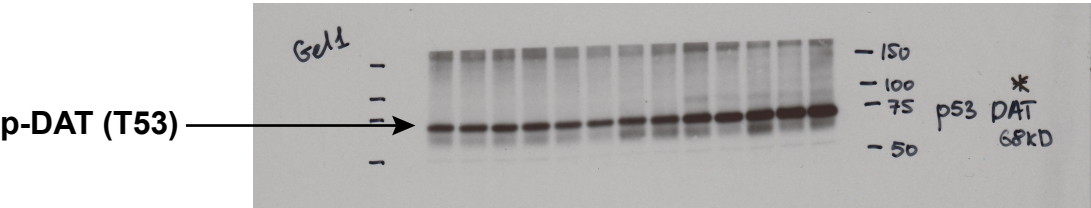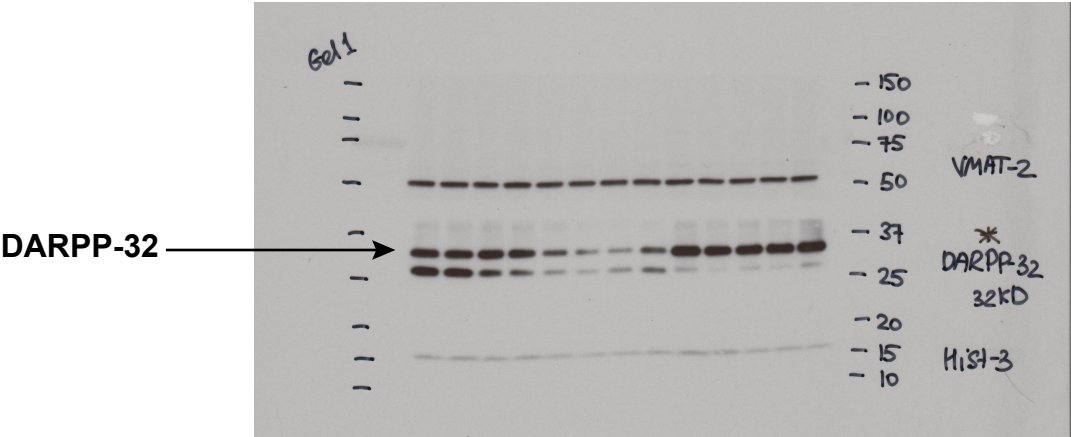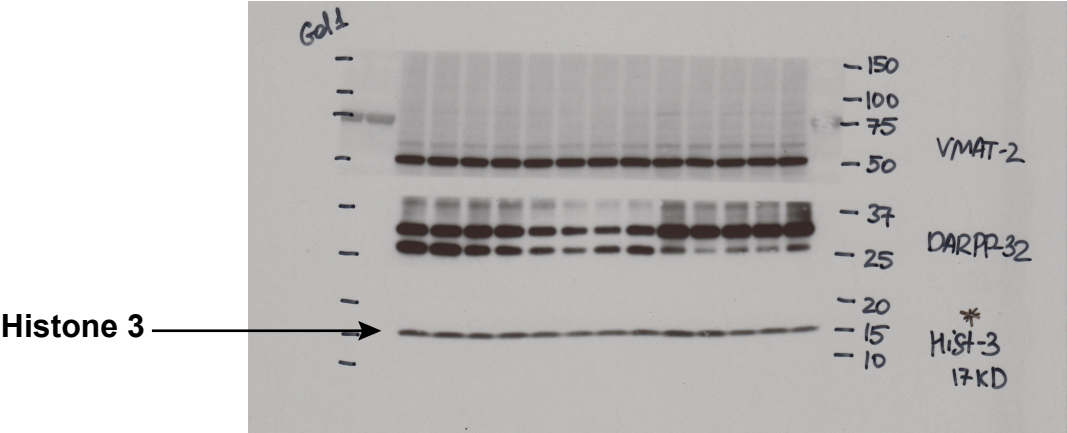

Supplement: Figure 9—source data 1. — The scans used for quantification for each antibody are indicated by the arrows. Films containing the first batch of samples (Gel 1) are shown. [file elife-75398-fig9-data1.pdf]

DA-Rictor KO  
Western blots  
Gel 2

VMAT-2

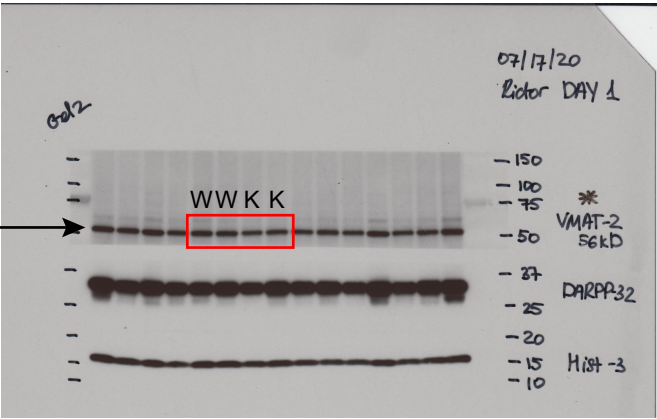

TH

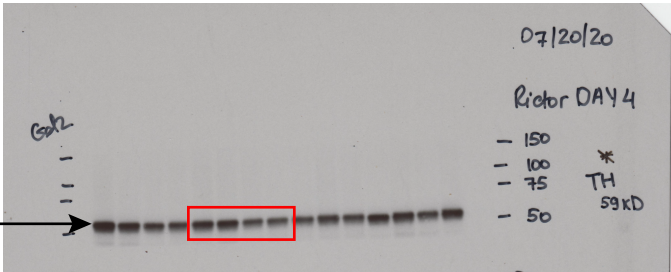

DAT

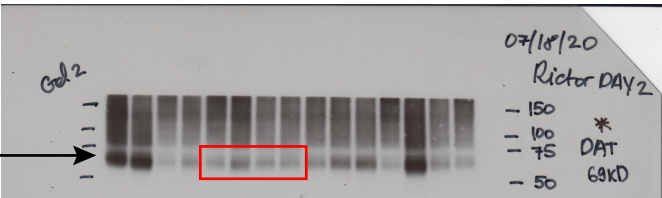

p-DAT (T53)

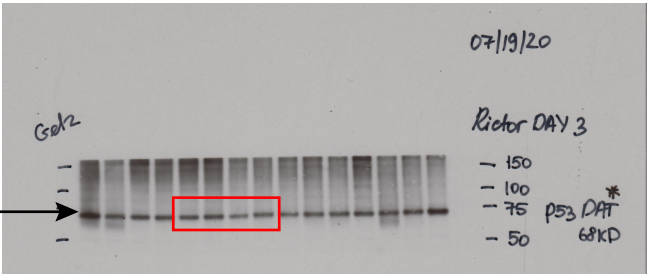

DARPP-32

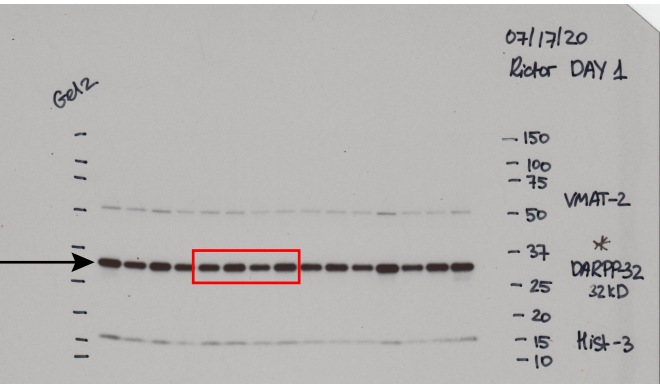

Histone 3

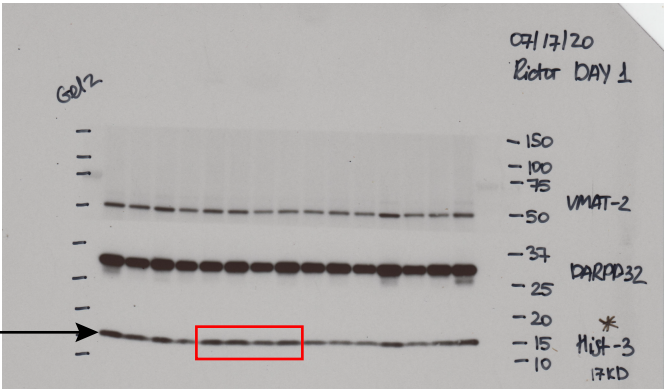

Supplement: Figure 9—source data 2. — The scans used for quantification for each antibody are indicated by the arrows. Films containing the second batch of samples (Gel 2) are shown. Shown in red are the cropped regions presented in Figure 9a. W=wild type, K=knock out. [file elife-75398-fig9-data2.pdf]
