# Supplementary material for: Dopamine neuron morphology and output are differentially controlled by mTORC1 and mTORC2
Source: eLife. 2022 Jul 26;11:e75398. doi: 10.7554/eLife.75398 (PMC9328766; doi:10.7554/eLife.75398)
Supplement: Figure 8—source data 3. [file elife-75398-fig8-data3.docx]

**Figure 8-Source Data 3. Raw values for HPLC measurements for DA-Raptor WT and KO mice, related to Figure 8.**

|  | **DA-Raptor WT** | | | | **DA-Raptor KO** | | | | **WT vs KO** |
| --- | --- | --- | --- | --- | --- | --- | --- | --- | --- |
| measurement | Mean | SEM | n (samples) | n (mice) | Mean | SEM | n (samples) | n (mice) | p-value/  test |
| Dorsal striatum  **DA**  (pmol/mm^3^) | 101.1 | 3.225 | 6 | 3 | 30.83 | 2.951 | 10 | 5 | **<0.0001** Welch’s t-test |
| Dorsal striatum  **DOPAC**  (pmol/mm^3^) | 2.573 | 0.4943 | 6 | 3 | 0.4440 | 0.0664 | 10 | 5 | **0.0073** Welch’s t-test |
| Ventral striatum  **DA**  (pmol/mm^3^) | 47.77 | 3.149 | 6 | 3 | 17.32 | 2.736 | 10 | 5 | **<0.0001** Welch’s t-test |
| Ventral striatum  **DOPAC**  (pmol/mm^3^) | 1.880 | 0.4212 | 6 | 3 | 0.6213 | 0.1722 | 8 | 4 | **0.0292** Welch’s t-test |
