# Supplementary material for: Dopamine neuron morphology and output are differentially controlled by mTORC1 and mTORC2
Source: eLife. 2022 Jul 26;11:e75398. doi: 10.7554/eLife.75398 (PMC9328766; doi:10.7554/eLife.75398)
Supplement: Figure 9—source data 3. [file elife-75398-fig9-data3.docx]

**Figure 9-Source Data 3. Raw values for HPLC measurements for DA-Rictor WT and KO mice, related to Figure 9.**

|  | **DA-Rictor WT** | | | | **DA-Rictor KO** | | | | **WT vs KO** |
| --- | --- | --- | --- | --- | --- | --- | --- | --- | --- |
| measurement | Mean | SEM | n (samples) | n (mice) | Mean | SEM | n (samples) | n (mice) | p-value/  test |
| Dorsal striatum  **DA**  (pmol/mm^3^) | 110.8 | 11.07 | 10 | 5 | 88.25 | 4.058 | 10 | 5 | 0.0809 Welch’s t-test |
| Dorsal striatum  **DOPAC**  (pmol/mm^3^) | 3.083 | 0.6679 | 10 | 5 | 2.170 | 0.3891 | 10 | 5 | 0.2566 Welch’s t-test |
| Ventral striatum  **DA**  (pmol/mm^3^) | 57.82 | 6.855 | 10 | 5 | 42.90 | 6.525 | 10 | 5 | 0.1323 Welch’s t-test |
| Ventral striatum  **DOPAC**  (pmol/mm^3^) | 2.541 | 0.4733 | 10 | 5 | 1.793 | 0.2639 | 8 | 4 | 0.2447 Welch’s t-test |
